# Left Atrial Myocardium in Arterial Hypertension

**DOI:** 10.3390/cells11193157

**Published:** 2022-10-08

**Authors:** Jens Kockskämper, Florentina Pluteanu

**Affiliations:** 1 Institute of Pharmacology and Clinical Pharmacy, Biochemical and Pharmacological Centre (BPC) Marburg, University of Marburg, 35032 Marburg, Germany; 2 Department of Anatomy, Animal Physiology and Biophysics, Faculty of Biology, University of Bucharest, 50095 Bucharest, Romania

**Keywords:** arterial (essential) hypertension, atrial myocardium, atrial myocytes, atrial remodeling, spontaneously hypertensive rats

## Abstract

Arterial hypertension affects ≈ 1 billion people worldwide. It is associated with increased morbidity and mortality and responsible for millions of deaths each year. Hypertension mediates damage of target organs including the heart. In addition to eliciting left ventricular hypertrophy, dysfunction and heart failure, hypertension also causes left atrial remodeling that may culminate in atrial contractile dysfunction and atrial fibrillation. Here, we will summarize data on the various aspects of left atrial remodeling in (essential) hypertension gathered from studies on patients with hypertension and from spontaneously hypertensive rats, an animal model that closely mimics cardiac remodeling in human hypertension. Analyzing the timeline of remodeling processes, i.e., distinguishing between alterations occurring in prehypertension, in early hypertension and during advanced hypertensive heart disease, we will derive the potential mechanisms underlying left atrial remodeling in (essential) hypertension. Finally, we will discuss the consequences of these remodeling processes for atrial and ventricular function. The data imply that left atrial remodeling is multifactorial, starts early in hypertension and is an important contributor to the progression of hypertensive heart disease, including the development of atrial fibrillation and heart failure.

## 1. Introduction—Clinical Significance of Arterial Hypertension

High blood pressure (arterial hypertension) poses a worldwide clinical and socioeconomic burden affecting ≈ 1 billion people and is responsible for millions of deaths each year [1,2,3]. Arterial hypertension is a risk factor for cardiovascular and renal disease, including devastating events like stroke or myocardial infarction [3,4,5]. Cardiovascular disease, in turn, is the leading cause of death worldwide. Cardiovascular alterations and disease associated with arterial hypertension (hypertension-mediated organ damage) include left ventricular (LV) hypertrophy, LV dysfunction and heart failure (HF) [3]. In addition, hypertension is also associated with left atrial (LA) enlargement, atrial cardiomyopathy and atrial fibrillation (AF) [3,6,7]. Thus, arterial hypertension affects not only the structure and function of the left ventricle but also causes LA remodeling, which, in turn, may impair LA function and predispose one to atrial arrhythmias. LA dysfunction and/or atrial arrhythmias contribute to LV dysfunction by causing impaired LV filling and the reduction of cardiac output.

In humans, essential or primary hypertension is, by far, the most common form of arterial hypertension, accounting for >90% of cases. The cause of primary hypertension is currently unknown. However, risk factors are known, such as age, male sex, smoking, high sodium intake, obesity or a positive family history [3]. Thus, essential hypertension represents a multifactorial disease with a genetic component. Usually, hypertension develops in adulthood, with the risk increasing with advancing age [3]. Uncontrolled or poorly controlled hypertension causes target organ damage, most notably affecting the vasculature, the heart, the kidneys and the brain, resulting in premature death [3,5]. Regarding the heart, arterial hypertension is associated with LV hypertrophy, ventricular and atrial arrhythmias, and HF [3]. In fact, hypertension is one of the leading causes of both AF and HF [8,9].

AF is the most common sustained arrhythmia. It is associated with increased morbidity and mortality, mainly resulting from thrombus formation in the LA appendage and subsequent stroke, as well as from impairment of LV function caused by reduced LV filling and irregular LV activation [10,11]. HF is associated with both arterial hypertension and AF. It may be divided into HF with reduced (HFrEF), mildly reduced (HFmrEF) or preserved ejection fraction (HFpEF) [8]. HF exhibits a high prevalence (1–2% in developed countries) and poor prognosis, with 5-year mortality rates in the range of 50% [8,12].

Antihypertensive drugs are used to treat hypertension (to reach target blood pressure) and to reduce hypertension-mediated organ damage and the risk of premature death [5]. Major classes of antihypertensive drugs include angiotensin-converting enzyme (ACE) inhibitors, angiotensin II (AngII) type 1 (AT_1_) receptor blockers (ARB), β-adrenergic receptor blockers (or β-blockers), Ca^2+^ channel blockers (or Ca^2+^ antagonists) and diuretics [3]. In addition, lifestyle and dietary changes are recommended for the treatment of arterial hypertension, such as the cessation of smoking, reduced sodium intake or weight reduction [3]. Thus, the treatment of hypertension targets known (modifiable) risk factors as well as the inhibition of the major neurohumoral systems involved in blood pressure regulation and the development of hypertension (see below).

## 2. Animal Models of Hypertension

Animal models of arterial hypertension represent invaluable tools for studying the pathogenesis of hypertension in general and of hypertension-mediated cardiac remodeling in particular, as patient data, specifically with regard to cellular and molecular remodeling of the heart, are limited. Moreover, they allow identification and validation of novel treatment targets. Nowadays, there are various animal models of hypertension available. These include rodent models and larger animal models, each with inherent advantages and disadvantages [13,14]. These animal models of hypertension are discussed in considerable detail in two recent and comprehensive reviews [13,14].

### 2.1. Spontaneously Hypertensive Rats, SHR

An animal model that recapitulates many features of essential hypertension in humans is the spontaneously hypertensive rat(s), SHR. SHR were developed in the 1960s by breeding Wistar rats which spontaneously developed hypertension [15]. Hypertension in SHR is of multifactorial etiology, including an activated stress axis, sympathetic nervous system (SNS) and renin-angiotensin-aldosterone system (RAAS) [14,16,17,18]. SHR are born normotensive and develop progressively increasing hypertension at 5–8 weeks of age [16,19]. Afterwards, they start exhibiting progressively increasing LV hypertrophy and LV fibrosis [20,21,22,23]. Finally, heart failure develops with features of both diastolic and systolic dysfunction [20,21,22]. SHR die prematurely [16]. Importantly, SHR also exhibit sex differences: females show less, more slowly developing hypertension and less LV hypertrophy, and they live longer than the males [20]. SHR are one of the few rodent models in which spontaneously occurring atrial tachyarrhythmias have been observed, the frequency of which increases with aging [24]. Finally, SHR respond to major classes of antihypertensives used to treat human essential hypertension [13]. Thus, SHR represent an important animal model, mimicking many aspects of human essential hypertension. This has led to the widespread use of these animals in basic and translational research. A PubMed (https://pubmed.ncbi.nlm.nih.gov/) search with the term “spontaneously hypertensive rats” accessed on 25 September 2022 yielded more than 23,000 results. Because of the many similarities between (cardiac remodeling in) human essential hypertension and SHR, we will focus on studies on SHR when discussing hypertension-induced LA remodeling.

### 2.2. Other Rodent Models of Genetic Hypertension

Genetic rodent models of essential hypertension in humans include Dahl salt-sensitive rats (DSS), fawn-hooded hypertensive rats, Lyon hypertensive rats, Milan hypertensive rats, Sabra hypertensive rats and Schlager hypertensive mice [14]. 

Dahl salt-sensitive rats (DSS) were selected from a colony of Sprague-Dawley rats that developed high blood pressure in response to high dietary salt intake [25]. In the same colony, some rats remained normotensive and did not develop cardiac hypertrophy with a high-salt diet. These rats were named Dahl salt-resistant rats and serve as controls for DSS. Selective breeding of these two colonies has led to the inbred rats currently used to investigate cardiac hypertrophy, renal dysfunction, endothelial dysfunction and genetics in hypertension. When fed a low-salt diet, DSS are normotensive until 6 weeks of age and then slowly develop hypertension with aging. Upon including 8% NaCl in the diet (high-salt diet), systolic blood pressure increased to 200 mmHg within 3 weeks, and the animals developed both atrial and ventricular myocyte hypertrophy, signs of heart failure, inflammation, and extensive apoptosis of cardiomyocytes. The median survival of DSS on a high-salt diet is 17 weeks, and they die mainly due to severe pulmonary infections with emphysema [26,27,28]. DSS are a model of low-renin hypertension and—unlike the SHR—they show resistance to treatment with ACE inhibitors and ARB [14]. There are only a few studies on atrial remodeling in DSS (see Table 1).

Regarding cardiovascular remodeling and, in particular, atrial remodeling, much less is known about the other genetic rodent models. Fawn-hooded hypertensive rats (FHH) emerged from the Long-Evans strain due to a spontaneous mutation that was further inbred in the Netherlands in the 1980s. These rats were initially considered a model for bleeding disorders. FHH develop hypertension spontaneously starting at around 5 weeks of age, reaching systolic blood pressure values of about 180–240 mmHg at 12 months of age [29]. Males develop hypertension earlier than females [30]. FHH are considered a genetic model for hypertension, chronic kidney disease and bleeding disorders and may also serve as a model for pulmonary artery hypertension [13,14]. Cardiovascular remodeling of FHH encompasses heart hypertrophy, myocardial fibrosis, myocardial infarction and generalized atherosclerosis [29]. FHH die from malignant nephrosclerosis or cardiac failure with chronic pulmonary congestion [29]. Milan hypertensive rats are a genetic model derived from Wistar rats. These animals exhibit spontaneous onset of hypertension at 7–8 weeks of age, and blood pressure further increases with aging [13]. Similar to the FHH, Milan hypertensive rats present a mutation in ADD3, a gene encoding for adducin, an actin-binding protein with a role in cytoskeletal organization that has been linked to hypertension in humans [31]. Lyon hypertensive rats were developed in France in the 1970s from a Sprague-Dawley strain and are considered a model of low-renin hypertension, similar to DSS and 11-deoxycorticosterone acetate (DOCA)-salt rats [14,32]. Lyon hypertensive rats are salt-sensitive, exhibit increased activity of the RAAS and hypersensitivity to AngII. The blood pressure increases spontaneously starting at 5 weeks of age, together with body weight and plasma lipids [33,34].

Even less is known about cardiovascular remodeling in Sabra hypertensive rats and Schlager hypertensive BPH/2J mice. Sabra hypertensive rats were selected as rats with high blood pressure after unilateral nephrotomy and DOCA salt treatment [35]. Schlager hypertensive BPH/2J mice are a model for neurogenic hypertension, being considered a genetic model for white-coat hypertension [14]. The mice display increased noradrenaline levels in the hypothalamus and amygdala, suggesting a centrally mediated hypertension [36,37]. Similar to SHR, Schlager hypertensive mice respond to treatment with ACE inhibitors [14]. 

### 2.3. Animal Models of Induced Hypertension

Experimental hypertension can be induced in a controlled manner in both small and large animals by targeting mechanisms known to be involved in blood pressure regulation and hypertension. Models include those mimicking RAAS activation (AngII model), endothelial dysfunction (N^ω^-nitro-L-arginine methyl ester (L-NAME) model), low-renin hypertension (DOCA-salt model), renovascular hypertension (1 kidney–1 clip, 1K-1C or 2 kidneys–1 clip, 2K-1C) or diet-induced models. Aspects of cardiovascular and renal remodeling have been investigated in all of these animal models. These experimental models of induced hypertension have in common a rather fast increase in blood pressure (developing within days to a few weeks), which is in contrast to human essential hypertension (developing over years) or the situation in SHR (hypertension developing over weeks to months).

The AngII model uses continuous infusion of AngII to induce hypertension. The model is used in both rats and mice. Blood pressure starts to increase already one day after the beginning of AngII infusion. Maximum values range from 160 to 200 mmHg. Over 2–4 weeks, animals display a linear increase of heart weight alongside an increase of blood pressure, vascular remodeling, cardiac hypertrophy and renal injury [38]. This model is relevant to RAAS-dependent hypertension, and it responds to treatment with ACE inhibitors and ARB (similar to SHR). Moreover, this model is characterized by cardiac hypertrophy, myocyte injury, perivascular infiltration, sympathetic hyperactivity and target organ damage (similar to SHR) [14]. At the cardiac level, both atrial and ventricular remodeling have been assessed. Some aspects of atrial remodeling have been addressed, including structural, electrical and inflammatory remodeling, as summarized in Table 1.

The L-NAME model targets NO-dependent vasodilation, an important endogenous mechanism involved in the endothelium-dependent regulation of blood flow. L-NAME is an NO synthase inhibitor. Long-term administration of L-NAME in the drinking water induces a progressive elevation of blood pressure to values of 160 to 200 mmHg after 4 weeks of treatment. This is a model of impaired endothelium-dependent vasorelaxation, leading to vasoconstriction, activation of the RAAS, oxidative stress and diastolic dysfunction, as well as renal and cardiac fibrosis [39,40]. Some aspects of atrial remodeling have been investigated, as shown in Table 1. The L-NAME model is easy to implement; it is reproducible, and hypertension can be reversed in response to NO donors or some antihypertensive drugs. Similar to the AngII model, blood pressure starts to increase rather quickly (within hours) in this model, and since this is different from the development of hypertension in humans, the L-NAME model is not considered an ideal model for essential hypertension in humans [13].

Experimental hypertension may also be induced by administration of DOCA and a high-salt diet. This is an affordable model that has been applied successfully to various species, including rats, mice, dogs, sheep and pigs. The DOCA-salt model is considered a mineralocorticoid-salt-dependent hypertension model characterized by sodium retention, volume increase and low circulating renin levels. Therefore, it is different from the other models introduced above, since the RAAS is suppressed in this model. Moreover, the DOCA-salt model does not respond to treatment with antihypertensive drugs. It is employed to understand hypertension in patients with primary aldosteronism and resistant hypertension. In this model, systolic blood pressure increases to 180 mmHg in rats [41] and to 140 mmHg in pigs [42,43]. DOCA-salt pigs are a model for HFpEF [42]. DOCA-treated animals develop cardiac hypertrophy, fibrosis and vascular dysfunction, along with sympathetic hyperactivation and inflammation. Some aspects of atrial remodeling have been studied in DOCA-salt rats or pigs, as summarized in Table 1. Other models of low-renin hypertension include 1- or 2-kidney 1-clip (1/2K-1C) rats or sheep [44,45]. In these models, the blood pressure increase stabilizes after a few weeks, and the animals develop LV hypertrophy with preserved LV function.

Table 1 lists some of the most frequently used animal models of hypertension and compares various levels of remodeling between these models, with particular emphasis on atrial remodeling.

## 3. Hyperactivity of the Sympathetic Nervous System Is a Hallmark of Essential Hypertension

The autonomic nervous system (ANS) controls the electrical and contractile activity of the atria, and activation or imbalance of the ANS in pathologies may be primarily associated with increased arrhythmogenicity. Atria receive both sympathetic and parasympathetic innervation, and stimulation of either component of the ANS may trigger atrial arrhythmias [46].

Upon activation of the SNS, postganglionic fibers originating from the stellate ganglion release noradrenaline in the heart. Patients with hypertension display signs of an activated SNS, including higher basal heart rate, increased blood pressure and elevated plasma catecholamines in early stages of hypertension [47,48,49,50]. Thus, sympathetic hyperactivity is currently considered a hallmark of essential hypertension [51]. Sympathetic hyperactivity has been reported for patients with borderline hypertension [52] or even in normotensive patients predisposed to develop essential hypertension [53]. Sympathetic hyperactivity may be induced by various mechanisms: an increased adrenergic response to environmental stimuli, impaired arterial baroreflexes, chemoreceptor stimulation, RAS activation and/or neurohumoral mediators [47]. Once initiated and if persistent, it will create a vicious circle causing chronic hypertension and hypertension-mediated organ damage.

In the heart, at the cellular level, noradrenaline (NA) stimulates the adrenergic receptors localized on atrial myocytes. β-adrenoceptor activation stimulates adenylyl cyclases (AC) via Gαs proteins to increase cAMP production and activate protein kinase A (PKA). PKA phosphorylates target proteins such as Cav1.2, ryanodine receptor type 2 (RyR2), phospholamban (PLB) and troponin I, eliciting positive inotropic and lusitropic effects. β-adrenergic stimulation also increases K^+^ currents such as the ultrarapid delayed rectifier, I_Kur_, and the slow delayed rectifier, I_Ks_ [10]. Due to a differential contribution of ionic currents to the action potential (AP), in human atrial myocytes, β-adrenergic activation causes shortening of the AP duration (APD) and the atrial effective refractory period (AERP), while in rodent models, catecholamines trigger increases in APD with an increased incidence of early afterdepolarizations (EADs). In both humans and rodents, there is a higher risk for (Ca^2+^-mediated) delayed afterdepolarizations (DADs) and ectopic activity, thus facilitating the development of arrhythmias. Prolonged β-adrenergic activation stimulates cAMP-dependent transcription, causing atrial enlargement and hypertrophy [54]. Thus, chronic β-adrenergic receptor activation of atrial myocardium may trigger Ca^2+^-dependent atrial arrhythmias and induce atrial remodeling. Moreover, activation of α_1_-adrenergic receptors by catecholamines may also induce atrial arrhythmias and atrial remodeling (via inositol 1,4,5-trisphosphate (IP_3_)-dependent Ca^2+^ release and/or mitogen-activated protein kinase activation) [55]. For example, NA increases SR Ca^2+^ leak and spontaneous Ca^2+^ release events in atrial myocytes and increases burst-induced AF duration in mice via activation of both α_1_- and β_1_-adrenergic receptors [56]. In addition, activation of α_1_-adrenergic receptors by phenylephrine mediates nuclear accumulation of nuclear factor of activated T cells (NFAT), which acts as a Ca^2+^-dependent transcription factor involved in cardiac remodeling, in an IP_3_-dependent manner [57]. In contrast to ventricular myocytes, which mainly express AC isoforms inhibited by Ca^2+^ (AC5 and AC6) [58,59], atrial myocytes show prominent expression of AC isoforms stimulated by Ca^2+^ (AC1, AC3 and AC8) [60,61]. This may have ramifications for Ca^2+^-dependent atrial arrhythmias and atrial remodeling through positive feedback between Ca^2+^ and cAMP-PKA signaling. In line with this notion, α1-adrenergic receptors may stimulate AC in atrial myocytes via IP_3_-induced SR Ca^2+^ release to increase cAMP-PKA signaling and atrial Ca^2+^ transients (CaTs) [62]. In summary, NA may induce atrial arrhythmias and atrial remodeling via various interdependent mechanisms involving activation of both α_1_- and β_1_-adrenergic receptors. 

Patients with hypertension also display signs of a reduction in the activity of the parasympathetic nervous system (PNS) [48]. Vagal innervation of the heart is particularly pronounced in the atria. Postganglionic parasympathetic fibers release acetylcholine (ACh) to activate muscarinic M_2_ receptors on atrial myocytes. M_2_ receptors signal via Gαi to inhibit AC, hence opposing β-adrenergic stimulation. In addition, M_2_ receptor activation, via Gβγ, activates I_K,ACh_, leading to shortening of the APD and the AERP [10]. Spectral analysis of the R–R interval in prehypertensive patients suggests an impaired vagal influence on the heart [48]. At later time points, early after the onset of hypertension, human patients manifest the first signs of high blood pressure and resting tachycardia, at reduced vagal drive, in line with the hypothesis that SNS activity progressively increases, while PNS remains at lower levels of activity [48].

In line with the altered ANS drive reported in human prehypertensive patients, increased N-type Ca^2+^ current, altered cyclic nucleotide signaling, larger Ca^2+^ transients and reduced NA reuptake were reported in the sympathetic stellate neurons of prehypertensive SHR, as early as 4 weeks of age [63,64]. Other studies reported an increased central sympathetic drive in SHR at 3 to 5 weeks of age [18] and changes in peripheral chemoreceptor sensitivity [65]. Moreover, carotid sinus denervation prevented the development of hypertension in young prehypertensive SHR and decreased arterial pressure in adult hypertensive SHR [66], as reported for hypertensive patients [67]. Collectively, these studies show that, already at the prehypertensive stage, there is a hyperactive SNS in SHR.

**Figure 1:**
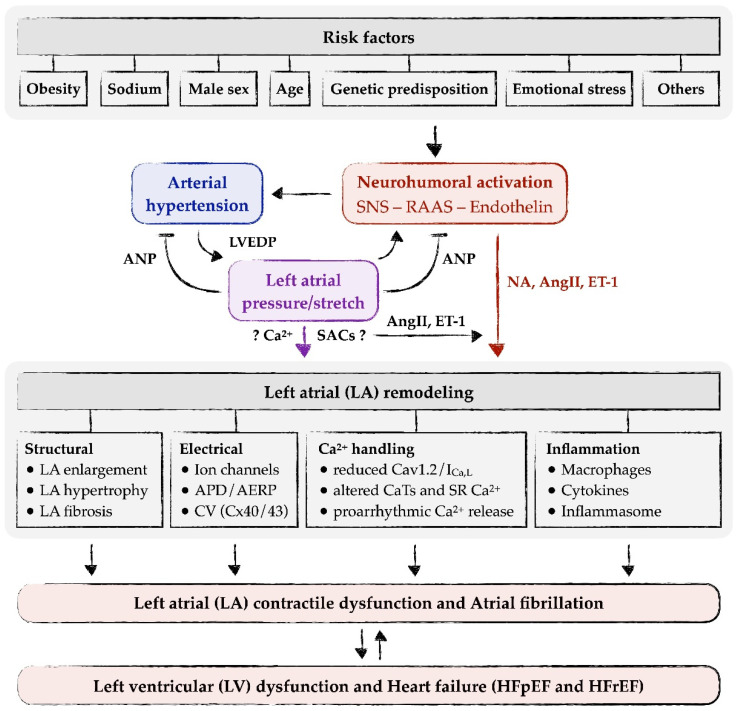
Risk factors for, initiating mechanisms of, LA remodeling in, and consequences of essential hypertension.

Thus, there is ample evidence that elevated activity of the SNS is an early event in both human essential hypertension and SHR (Table 1), presumably initiating the development of arterial hypertension as well as atrial (and ventricular) remodeling (Figure 1).

## 4. The Left Atrium

### 4.1. Clinical Significance of the Left Atrium

The left atrium serves important physiological functions: it collects and stores the oxygenated blood from the pulmonary veins during LV systole (reservoir function), transmits the blood into the left ventricle during early LV diastole (conduit function), and LA active contraction increases LV filling during LV diastole (booster pump function) [6]. Thus, LA function is an important determinant of LV function and cardiac output. The left atrium is innervated by the ANS, which modulates LA electrical and contractile activity. Altered activities of either the SNS or the PNS may trigger atrial arrhythmias. The pulmonary veins, which lead into the left atrium, often act as initiation sites of AF and the left atrium may be a driver of AF in many patients [68,69,70]. Hence, isolation of the pulmonary veins has become a mainstay of AF therapy [70]. In AF, contractile function of the atria is lost, leading to thrombus formation, in particular in the LA appendage, with consequences for treatment (anticoagulation or closure of the LA appendage). Moreover, the loss of the booster pump function in AF impairs LV function and contributes to the high prevalence of HF in AF patients [10]. Even in the absence of AF, impaired LA function compromises LV function and is associated with adverse outcomes [71]. Hypertension is present in many patients with AF and/or HF and hypertension-mediated changes in LA structure and function, i.e. LA remodeling, is thought to underlie or facilitate the development of AF and HF [8,10,11]. Therefore, it is of utmost importance to study LA remodeling in hypertension and to better understand the time course, the underlying mechanisms and the consequences of these remodeling processes.

### 4.2. How Hypertension May Affect the Left Atrium (and Vice Versa)

Arterial hypertension may affect the left atrium via two main mechanisms: (1) hemodynamic and (2) neurohumoral. When arterial hypertension develops, it first poses an increased afterload on the left ventricle, which is forced to develop higher pressures to pump the blood into the circulation. This results in higher LV filling pressures, which, in turn, may increase the pressure in the left atrium. Increased LA pressure causes increased atrial wall stress. The atria are particularly vulnerable to this form of stress, as they usually deal with low pressures (well below 20 mmHg) and have thin walls (a few millimeters) [6,72]. Elevated LA pressures have been observed in patients with arterial hypertension [2] as well as in animal models of hypertension, notably also in the SHR [73]. Moreover, elevated LA pressure and/or atrial stretch are known triggers for atrial remodeling and AF [74]. 

Stretch of isolated atrial myocytes or the atrial myocardium has three major effects: (1) an immediate increase of atrial contractile force via the Frank-Starling mechanism, (2) an immediate increase in SR Ca^2+^ release (Ca^2+^ sparks) and (3) the release of hormones such as natriuretic peptides (atrial natriuretic peptide, ANP, and B-type natriuretic peptide, BNP), AngII and endothelin-1 (ET-1) [75,76,77]. Atrial myocytes appear to be the sole source of natriuretic peptides, whereas AngII and ET-1 may be released by various cell types of the atrial wall, including endocardial cells, fibroblasts and atrial myocytes [78,79]. AngII and ET-1 mediate both short- and long-term effects on atrial function and remodeling, as outlined below.

Neurohumoral activation, i.e., increased activity of the RAAS, the endothelin system or the SNS, is often associated with arterial hypertension, including SHR [2,64,80,81]. This may either be a cause of hypertension itself or the result of an adaptation of the heart to the increased load imposed by the increased blood pressure. Mediators of these systems, e.g., AngII, aldosterone, ET-1 or NA, induce or contribute to atrial remodeling [80,81,82,83,84]. Long-term effects on atrial remodeling include atrial dilatation/hypertrophy, atrial fibrosis, atrial electrical remodeling and other forms of remodeling (see below and Figure 1). In addition to their long-term effects on atrial remodeling, AngII and ET-1 may also exert acute, direct effects on the atrial myocardium or atrial myocytes. These acute effects include increased CaTs and contractility, pro-arrhythmogenic Ca^2+^ release and extra-contractions, and elevated nuclear Ca^2+^ [55,75,85,86,87,88,89]. Increased CaTs may enable the atrial myocardium to adapt atrial contractility to the elevated load, pro-arrhythmogenic Ca^2+^ release may trigger atrial arrhythmias and contribute to the increased risk of AF, and elevated nuclear Ca^2+^ may contribute to long-term atrial remodeling via alterations in transcription (excitation-transcription coupling).

Autocrine/paracrine release and action of AngII and ET-1 (and possibly other mediators) by elevated LA pressure/stretch may explain most of the LA remodeling processes in hypertension (Figure 1). In addition, most of the signaling pathways and cellular mechanisms involved in LA remodeling can be linked to either AngII or ET-1 signaling (see Section 5 on Left atrial remodeling in arterial hypertension). The question arises whether there are other, more direct means by which LA pressure/stretch may induce LA remodeling (Figure 1, purple arrow). Since stretch induces an immediate increase in SR Ca^2+^ release in atrial myocytes [76], it could be speculated that altered intracellular Ca^2+^ signaling contributes to LA remodeling, e.g., via altered excitation-transcription coupling. In HL-1 atrial myocytes, stretch causes reduced expression of SERCA and Ca^2+^ alternans [90]. Moreover, stretch activates cation channels in the cell membrane (stretch-activated channels, SACs), which may alter intracellular Ca^2+^ via alterations in membrane potential and/or via influx of Ca^2+^ and Na^+^. However, these putative “direct” effects on remodeling are difficult to discern from the “indirect” effects of AngII or ET-1, as these mediators might be released from the cells at the same time. This is best exemplified by a study in an isolated cell system, where axial stretch of a single isolated (ventricular) myocyte caused a slow increase in CaTs that was sensitive to blockers/knockout of (stretch-dependent) transient receptor potential canonical (TRPC) channels and ARB, suggesting that stretch-dependent release and autocrine action of AngII mediated the increase in CaTs [91]. Thus, although it remains feasible that there are more direct effects of LA pressure/stretch on LA remodeling, sound experimental evidence for this is scarce.

Hypertension-mediated atrial remodeling is not a one-way street, in which mechanical and neurohumoral factors act on the atria to induce remodeling, but rather the atria themselves may act on the periphery to regulate blood pressure. The atria are central to volume regulation and act as an endocrine organ. Upon an increase in volume or stretch, atrial myocytes in the atria (both right and left) release natriuretic peptides, ANP and BNP [77,92]. Natriuretic peptides, in turn, act on the kidneys and the vasculature to promote natriuresis, diuresis and vasodilation [93], with the combined effect being a reduction in volume and blood pressure. Moreover, natriuretic peptides also inhibit both the SNS and the RAAS [93], thus counteracting their blood pressure-raising effects. In SHR, the infusion of ANP caused a large reduction in mean arterial pressure (≈40 mmHg), significantly larger than in normotensive Wistar-Kyoto rats (WKY) [94]. Similar observations were made in another rat model of hypertension [95]. Moreover, the natriuretic and diuretic effects of ANP were smaller in SHR than in WKY [94]. Under basal conditions, plasma ANP levels in SHR were very similar to those found in normotensive WKY [96]. An acute volume load increased plasma ANP levels in both WKY and SHR, but the maximal response in SHR was blunted [96]. These results show that ANP has potent blood pressure-reducing (and natriuretic/diuretic) effects, and these are qualitatively preserved in hypertension. However, in SHR, both the volume/stretch-induced release of ANP from the atria and the effects of ANP in the periphery are blunted, suggesting an impaired ability of the natriuretic peptide system to antagonize the SNS and the RAAS.

More recently, a study on patients with AF (all of them with hypertension) undergoing left atrial appendage (LAA) closure revealed evidence for another (novel) mechanism by which the left atrium may act back on neurohumoral systems to regulate blood pressure [92,97]. The study showed that when LAA closure is achieved by an epicardial closure procedure (using the Lariat device), which leads to subsequent atrophy and fibrosis of the LAA [92], there is profound and persistent (3 months) reduction in blood pressure (by ≈15 mmHg for systolic and ≈10 mmHg for diastolic blood pressure). The blood pressure reduction was associated with decreases in plasma levels of adrenaline, noradrenaline, aldosterone and renin [97]. A subsequent study with patients with persistent AF (80% with hypertension) confirmed these findings and showed that blood pressure reduction and decreases in adrenaline, noradrenaline, aldosterone, renin and vasopressin persisted for up to 2 years post LAA closure [98]. These results suggest that the LAA modulates other neurohumoral systems by yet-to-be-defined mechanisms and that (functional) removal of the LAA causes long-lasting inhibition of the SNS and the RAAS (and vasopressin). They emphasize that the atria may exert additional blood pressure–modulating effects beyond natriuretic peptide release.

## 5. Left Atrial Remodeling in Arterial Hypertension

### 5.1. Structural Remodeling

At the macroscopic level, the first evidence for structural remodeling induced by hypertension is LA enlargement. Various methods may be used to detect and evaluate the increase in atrial size in human patients and animal models. A simple and easy (albeit gross) means to detect atrial enlargement is the electrocardiogram (ECG), where increased duration of the P-wave is indicative of enlarged (left) atria [99]. In clinical practice, however, more useful methods to assess LA size, structure and function in sufficient detail are echocardiography, cardiac computed tomography and cardiac magnetic resonance imaging [6]. LA enlargement in hypertension has been reported for patients with arterial hypertension [100,101,102] as well as for animal models of hypertension, such as SHR [23,103,104,105] or hypertensive pigs [43], mostly in advanced hypertension during the transition to heart failure. Interestingly, in hypertensive patients, LA enlargement may occur before the development of LV hypertrophy, suggesting that it may be a rather early event [102]. Clinically, left atrial size is an important parameter, as increasing LA volume is associated with hypertension and cardiovascular outcomes such as LV structural and functional abnormalities and mortality [106].

Multiple alterations on the molecular, cellular and tissue level may be responsible for LA enlargement in hypertension. In addition to atrial myocytes, the atrial walls contain various other cell types, such as fibroblasts, adipocytes or inflammatory cells, blood vessels and non-cellular components, e.g., collagen fibers [6]. LA enlargement, therefore, could be the result of an increase of atrial myocyte size (hypertrophy), an increase of the total number of cells or an increase of extracellular collagen (fibrosis). Limited information on microscopic (histological and ultrastructural) changes in atrial biopsies from hypertensive patients (with no further comorbidities) is available. Therefore, most of the current knowledge is provided by studies performed in animal models of hypertension.

Mechanistically, an increased number of fibroblasts results from fibroblast activation, either compensatory due to a lesion (reparative fibrosis after myocyte apoptosis or necrosis) or as a result of the presence of profibrotic factors, e.g., proinflammatory cytokines or activation of the RAAS or the endothelin system [2,80,81,107,108]. Importantly, fibroblasts are activated by stretch and release ET-1 [79]. Fibroblast activation will trigger fibroblast proliferation and differentiation to myofibroblasts and, in the long term, the secretory phenotype will increase the amount of collagen in the extracellular matrix, therefore leading to fibrosis. 

Studies on animal models have helped delineate the time course of atrial fibrosis during hypertension. Experiments on SHR have shown that LA fibrosis appears as early as 3 months of age [104], with evidence of increased epicardial and interstitial fibrosis from 4 to 24 months of age [104,105,109,110,111]. Moreover, there was a progressive increase of atrial mRNA levels of collagen 1a, 3a and connective tissue growth factor (CTGF) with progression of LV hypertrophy, while the increase of atrial transforming growth factor-β (TGFβ) and matrix metalloproteinase 2 coincided with LA dilatation and the transition from compensatory hypertrophy to heart failure [103,109]. Increased atrial TGFβ supports a robust inflammatory component that may further contribute to the formation of fibrosis [81,103], while matrix metalloproteinase 2 allows for infiltration of inflammatory cells or migration of activated fibroblasts [103], both potentially contributing to LA enlargement.

In SHR, LA myocyte size was not altered (compared to age-matched WKY) at any stage of the disease when assessed in isolated myocytes, either by the 2D area of the cells (microscopy) or by membrane capacitance (patch clamp) [103,104,109]. Histological analysis of the left atria from 9–12 months old SHR, however, revealed a 50% increase of the cross-sectional area of LA myocytes (compared with age-matched WKY) [110]. Interestingly, the same study showed that the atrial myocyte hypertrophy (and fibrosis) may be reversed by exogenous administration of the hormone relaxin, even when applied at the late stages of chronic hypertension [110]. Similar results of increased cross-sectional area of LA myocytes have been reported from larger animal models, such as pigs treated with DOCA and fed a high-glucose/salt/potassium diet, at time points when left atria displayed reduced contractility, no increased fibrosis, and moderate LV hypertrophy with preserved ejection fraction [43]. In a rat model of elevated afterload, atrial myocyte hypertrophy was also observed [112]. In a study on patients without a history of AF, heart failure or cardiomyopathy undergoing cardiac surgery for various reasons, 72% of patients exhibited hypertension. Histological analysis of LA biopsies indicated only the presence of focal areas of myocyte hypertrophy [113]. Collectively, these results support the notion that LA myocyte hypertrophy may occur during established hypertensive heart disease, but that it does not represent an early event. Among the mechanisms potentially involved in LA myocyte hypertrophy, mechanical stretch and neurohumoral factors such as AngII, ET-1, or catecholamines are likely candidates.

In summary, LA enlargement, as a hallmark of atrial structural remodeling, is a macroscopic structural alteration accompanied by other remodeling processes (such as fibrosis and atrial myocyte hypertrophy) in the atria. Many studies have shown that LA enlargement occurs rather late in hypertensive heart disease, when much of the remodeling is considered irreversible. Studies on animal models, however, suggest that, at the molecular level, atrial structural remodeling may start soon after the onset of hypertension. Therefore, because of its early development, contribution to disease progression and potential reversibility, LA structural remodeling represents an attractive treatment target in essential hypertension. Future studies should focus on answering the following questions: (1) When exactly does (a particular form of) LA structural remodeling start in which model and in which hypertensive patients with which comorbidities? (2) What is the time course of progression (of which form) of LA structural remodeling? (3) Is there a particular time point when a particular form of LA structural remodeling becomes irreversible? New or refined noninvasive techniques might be required to answer these questions, in particular for patients with hypertension.

### 5.2. Electrical Remodeling

The cardiac electrical activity can be noninvasively recorded by electrocardiography (ECG). Atrial electrical activity is estimated from the duration and amplitude of the P wave and the P-R interval. In 1963, G. Ross conducted one of the first studies in which properties of the P wave were correlated with various forms of hypertension (including hypertension with diastolic pressures < or >120 mmHg, malignant hypertension and hypertensive failure) [114]. In hypertensive patients, P wave duration and amplitude were augmented. Moreover, the incidence of notched or bi-peak P waves was increased in more severe hypertension and hypertensive heart failure, suggesting LA hypertrophy [114,115]. On the other hand, the P-R interval of patients with hypertension was unaltered, suggesting unaltered atrioventricular conduction [114]. P wave changes have also been reported in animal models of hypertension, such as SHR, where progressive increases of the P wave duration and the incidence of bi-peak P waves were detected with advancing age [23]. Of note, in contrast to early ECG recordings from hypertensive patients [114], ECG recordings from SHR showed a progressive, age-dependent increase in the duration of the P-R interval, suggesting impaired atrioventricular conduction [23].

Electrical conduction within the atria mainly depends on electrical coupling of atrial myocytes (through gap junctions) and tissue fibrosis. Increased fibrosis has been demonstrated in atria from SHR (as discussed above) and in other rat models of elevated afterload, e.g., [116]. Atrial gap junctions are mainly composed of connexins (Cx) 40 and 43. While altered expression and/or location of connexins has been observed in human and rat atria in AF [117,118], connexin expression has yet not been studied in detail in atria from hypertensive patients or SHR, although one study reported reduced phosphorylation and another reduced expression of Cx43 in SHR atria [110,119]. Similarly, in a rat model with elevated afterload, expression of Cx43 was greatly reduced [116].

Reduced expression of connexins is expected to decrease intra-atrial conduction velocity. In SHR, decreased atrial conduction velocity was observed at 9–12 months of age [110], and increased atrial conduction heterogeneity at 12–15 months of age [105]. Decreased atrial conduction velocity and/or increased conduction heterogeneity were also reported from a rat model of elevated afterload [116] and from larger animal models of hypertension [120,121,122]. Finally, patients with long-standing hypertension also exhibited decreased conduction velocity [123]. Importantly, reduction of blood pressure in patients with resistant hypertension that underwent renal denervation increased conduction velocity [124] suggesting reversibility of the underlying atrial remodeling.

APD is an important readout for electrical remodeling. APD determines the refractory period and affects the inducibility of arrhythmias. A hallmark of AF, for example, is shortening of the atrial APD and refractory period, which facilitates reentry and perpetuation of the arrhythmia [10]. Little is known, however, about the atrial action potential characteristics of patients with essential hypertension (and no further comorbidities). In SHR, however, monophasic action potentials (MAP) have been recorded from left atria at 3 and 11 months of age [104]. Another study assessed APD by a voltage-sensitive fluorescent dye in the left atria from Langendorff-perfused hearts of 9–12-month-old SHR [110]. In both studies, no major changes in the left atrial APD or in the atrial effective refractory period were observed in SHR [104,110]. At variance with this, a recent study reported increased APD in atrial myocytes from 6-month-old SHR [119]. By contrast, in older SHR (12 to 15 months) the LA effective refractory period was smaller than in age-matched WKY controls, suggesting that the atrial APD may be shortened [105].

Despite unchanged atrial MAP duration in SHR up to 11 months of age, several changes in sarcolemmal ion currents were observed. Steady-state and peak outward K^+^ currents (presumably I_to_) were moderately increased at 11 months of age [104]. Another study, however, found reduced outward K^+^ currents along with reduced expression of Kv1.5 and prolonged APD [119]. Additionally, atrial L-type Ca^2+^ currents (I_Ca,L_) were decreased in SHR as early as 3 months-of-age and reduced expression of the α1C subunit (Cav1.2) was found at 6 months-of-age [104,109]. At the same time, the current carried by the Na^+^/Ca^2+^ exchanger (NCX), which is an inward (depolarizing) current during most of the action potential, was decreased despite unaltered protein expression [109]. Finally, the late Na^+^ current (I_Na,late_) was increased in atrial myocytes from SHR [125]. Thus, there is considerable remodeling of ion currents in atrial myocytes from SHR, and this may affect intracellular ion handling (Na^+^, Ca^2+^) and propensity for arrhythmia.

### 5.3. Ca^2+^ Handling Remodeling

In cardiomyocytes, Ca^2+^ is essential for mediating contraction during excitation–contraction coupling. It also serves other pivotal functions, such as regulating mitochondrial metabolism or transcription. Disturbed cardiomyocyte Ca^2+^ handling is a hallmark of many cardiac diseases. Reduced cytosolic CaTs will reduce contractions, and diastolic SR Ca^2+^ release may trigger arrhythmias.

Ca^2+^ handling has been studied in atrial myocytes from SHR starting at 3 months of age up to 25 months of age, i.e., from the early stages of hypertensive remodeling until the manifestation of heart failure [103,104,109]. The first sign of altered Ca^2+^ handling was reduction of I_Ca,L_ at 3 months of age [104]. Reduced I_Ca,L_ was also observed at later time points and accompanied by reduced expression of the α1C subunit (Cav1.2) [103,104,109]. It resulted in reduced subsarcolemmal Ca^2+^ increases [109]. At 6 months of age, further alterations in Ca^2+^ handling became obvious, including altered expression and phosphorylation of RyR2, reduced activity of NCX (at unaltered intracellular [Na^+^]) and augmented SR Ca^2+^ load [109]. CaTs, however, were still mostly unchanged at this stage, suggesting that the observed Ca^2+^ remodeling could largely preserve CaTs and contractions. At higher stimulation frequencies, however, an increased incidence of pro-arrhythmic Ca^2+^ alternans occurred, suggesting an increased risk for the development of Ca^2+^-dependent arrhythmias already at this rather early stage of hypertensive heart disease.

In advanced hypertensive heart disease and in heart failure, further Ca^2+^ handling remodeling was observed in SHR atrial myocytes [103]. CaTs showed increases in diastolic [Ca^2+^] and slowing of CaT decay with the development of heart failure. SR Ca^2+^ load was increased, but fractional SR Ca^2+^ release greatly diminished in heart failure. Increased propensity for proarrhythmic Ca^2+^ alterations (Ca^2+^ alternans, spontaneous Ca^2+^ increases) was also observed. These alterations of atrial myocyte Ca^2+^ handling were associated with reduced expression of α1C (Cav1.2), RyR2 and calsequestrin and progressively decreasing expression of SERCA2a, indicating severe impairment of SR Ca^2+^ handling. Interestingly, in heart failure, phosphorylation of RyR2 (and PLB) was decreased at sites predominantly phosphorylated by Ca^2+^/calmodulin-dependent protein kinase II (CaMKII) and unchanged at sites predominantly phosphorylated by PKA. This is in clear contrast to ventricular Ca^2+^ handling remodeling observed in most heart failure models (including humans and SHR), which exhibit increased phosphorylation of RyR2 at both CaMKII and PKA sites [126,127]. Moreover, ventricular myocardium from SHR with heart failure features increased (rather than decreased) CaTs [128]. These findings highlight that there may be differential remodeling (of Ca^2+^ handling) between LA and LV myocardium in hypertensive heart disease and heart failure. 

In DOCA-treated pigs with hypertensive heart disease, LA myocytes exhibited decreased CaTs with prolonged decay and reduced SR Ca^2+^ load [43]. Expression of Ca^2+^ handling proteins, however, was not assessed in that study. 

Thus, altered LA Ca^2+^ handling appears to be a common and rather early form of remodeling in hypertension, although more data is required from other animal models and, in particular, from hypertensive patients. Atrial Ca^2+^ handling remodeling contributes to the progression of hypertensive heart disease, is probably involved in the transition to heart failure, and may be a trigger for atrial tachyarrhythmias. Hence, normalization of atrial myocyte Ca^2+^ handling should be a primary future treatment goal in essential hypertension.

### 5.4. Pro-Inflammatory Remodeling

There is increasing evidence for inflammation or pro-inflammatory remodeling in hypertension. Whether this inflammation is a consequence or a cause of hypertension, however, is still unclear. Studies in various models of hypertension (some shown in Table 1) as well as observations from patients with essential hypertension indicate that inflammation is an important component of the pathogenesis. Patients with hypertension show either increased levels of pro-inflammatory cytokines, such as tumor necrosis factor α (TNFα), interleukin (IL)-1β, IL-6, IL-8 and IL-17, or increased production capacity, consistent with the existence of activated monocytes in the circulation and in line with the potential existence of a low-grade inflammatory state in prehypertensive patients [129,130]. The close relationship between inflammation and hypertension is reflected by the decrease of pro-inflammatory cytokines and the increase of the anti-inflammatory IL-10 caused by antihypertensive treatment with ARB or Ca^2+^ channel blockers [129,131]. On the other hand, anti-inflammatory treatment in hypertension was able to reduce hypertension-related complications, to prevent the onset of hypertension or to reduce blood pressure [132,133,134]. These studies point to a role of inflammation in the pathogenesis and progression of hypertension. 

Is the inflammation systemic or local? Any cardiac insult can initiate an inflammatory response in the heart. The mechanical stretch of the atrial or ventricular wall in hypertension may constitute such an insult. Different models of hypertension may display different types of inflammation: experimentally induced hypertension may show acute cardiac inflammation mediated by oxidative stress, which results in a rapid decline of cardiac function, while genetic hypertension models may display chronic inflammation, which develops more slowly and may cause progressive structural damage, leading to cardiac fibrosis [135]. The mediators of inflammation are of cellular and humoral nature, and both types may be systemic (circulating) or locally active. 

Are the atria prone to inflammation? At the cellular level, cardiac tissue contains various cell types, including cardiomyocytes, fibroblasts and immune cells. Interestingly, the fraction of immune cells is much larger in atrial (10%) than in ventricular (5%) tissue, suggesting that the atria are more prone to the development of local inflammation [136]. Among the immune cells, macrophages are resident cells, while monocytes are likely circulating cells [136]. The atria can be exposed to inflammatory mediators originating from at least three different sources: (1) circulating cytokines, (2) mediators locally released by the pro-inflammatory phenotype of activated macrophages, fibroblasts or cardiomyocytes, and (3) mediators released locally by infiltrating immune cells following myocardial insult [137]. 

How could hypertension activate inflammation in the atria? One mechanism may involve the increased (local and global) AngII levels in hypertension. AngII may activate both circulating T-cells and resident macrophages to produce and secrete chemokines and cytokines via signals involving reactive oxygen species (ROS) and further activation of the nuclear factor κB (NFκB) [138]. Once the resident macrophages are activated, the inflammatory environment will be reinforced by inducing a pro-inflammatory phenotype in fibroblasts and cardiomyocytes, and also by recruiting circulating monocytes to the site of injury. Upon NFκB activation, both pro-inflammatory fibroblasts and cardiomyocytes will synthesize and secrete cytokines: TNFα, IL-1β and IL-6. Macrophages may stimulate cardiac fibroblasts to produce IL-6, and IL-6, in turn, is essential for TGFβ-induced cardiac fibrosis [139]. In atria from 4-month-old SHR, there is increased expression of AT_1_ receptors and TGFβ associated with increased fibrosis [111]. Left atria from 6-month-old SHR exhibit elevated expression of TNFα, IL-1β, IL-6 and TGFβ, along with increased fibrosis and inducibility of AF [119]. Strikingly, the left atria from these animals also show increased expression of the pro-inflammatory chemokine CXCL1 and its receptor CXCR2, and inhibition of CXCR2 reduces the elevated levels of the cytokines, the LA fibrosis and the inducibility of AF [119]. In HL-1 atrial myocytes, elevated pressure upregulated pro-inflammatory cytokines (TNFα) and stretch caused recruitment of macrophages via ATP release [140,141]. Once recruited from the circulation to the site of chemokine release, the immune cells release ROS and proteases as a defense mechanism, facilitating further infiltration. Increased atrial oxidative stress has been reported in left atria of 6–8-month-old SHR, in AngII-infused mice, in DSS and in DOCA-salt pigs at time points when atrial fibrosis as well as pro-inflammatory and pro-fibrotic cytokines were increased [28,81,119,142]. Notably, while for the AngII and DSS model, infiltration of macrophages was detected at the same time point, in SHR this occurred only at ≥12 months of age [105]. NFκB activation was reported in left atria of SHR and in DOCA-salt rats [81,119,143]. Collectively, these studies demonstrate that all the mediators required to initiate inflammation are present in atria from various animal models of hypertension. 

Cytokine production by myocytes and fibroblasts can be triggered not only by ROS/NFκB activation but also following the activation of the innate immune system. The innate immune system emerged as an important determinant of hypertension and end-organ damage via pathways involving Toll-like receptors (TLR) and the inflammasome; both increased in 5-month-old SHR and in DOCA-salt rats after 12 weeks of treatment [143,144]. Inflammasomes are intracellular multiprotein complexes; the best understood is the nucleotide-binding oligomerization domain (NOD)-like receptor containing pyrin domain 3 (NLRP3). Downstream NLRP3, caspase-1 cleaves (and thereby activates) the proforms of the pro-inflammatory cytokines IL-1β and IL-18 prior to secretion [145,146]. These cytokines will attract immune cells to the infection site, initiating the inflammatory cell death pathway [146]. In the absence of pathogens, inflammasomes can be activated by various endogenous factors, including ROS and extracellular ATP [146].

Overall, these studies suggest that atrial inflammation may occur in hypertension, most probably caused by activation of the RAAS and further stretch-dependent release of AngII (and ET-1) and cytokines as well as recruitment of immune cells. The elevated levels of AngII increase ROS production and activation of cytokine production in resident macrophages, fibroblasts and cardiomyocytes, via NFκB or NLRP3 inflammasome activation. The local inflammation will promote atrial structural remodeling, mainly by increasing fibrosis via TGFβ, and it will also affect atrial electrical remodeling [119]. The relationship between immune remodeling and electrical remodeling in atria and atrial myocytes has been recently reviewed [147]. Key ion currents/channels and transport proteins affected by cytokines are: I_Ca,L_ (reduced by TNFα, IL-1β), I_to_ (increased by TNFα), SERCA2a (reduced by TNFα), RyR2 (increased following NLRP3 activation) and Cx43 (decreased by IL-6). Some—but not all—of these alterations are also found in the atrial myocytes of SHR. Future studies should aim at better understanding the crosstalk between the immune system and the atrial myocardium and clarifying whether the link between immune cells and atrial remodeling in hypertension can be exploited therapeutically by specifically targeting cytokines/chemokines and immune cells.

In summary, as illustrated in Figure 1, LA remodeling in essential hypertension is initiated and driven by two interdependent mechanisms: neurohumoral activation (with early hyperactivity of the SNS) and elevated LA pressure/stretch. The latter reinforces neurohormonal activation by local release of AngII and/or ET-1. Remodeling consists of various alterations in structure and function of the left atrium, including LA enlargement, myocyte hypertrophy, fibrosis, decreased conduction velocity, altered Ca^2+^ handling and inflammation. Consequences of these remodeling processes are LA contractile dysfunction and augmented risk for development of atrial arrhythmias, notably AF, as outlined below.

## 6. Consequences of Left Atrial Remodeling in Hypertension

### 6.1. Left Atrial Contractile Dysfunction

The structural, electrical, Ca^2+^ handling and pro-inflammatory remodeling described above is expected to impair or reduce LA contractile function in the long term. Increased LA fibrosis will stiffen the left atrium and impair LA filling and contraction. Slowed or heterogeneous atrial electrical conduction might affect coordinated atrial contractions. Elevated diastolic [Ca^2+^]_i_ and prolonged CaT decay impair atrial (myocyte) relaxation. Even if CaT amplitude is maintained (as observed in SHR atrial myocytes), this might not suffice for efficient LV filling in the presence of elevated LV end-diastolic pressure (LVEDP). In addition, CaT alternans will cause alternating degrees of LV filling. 

In line with these considerations, several studies indicated atrial contractile dysfunction in hypertensive patients as well as in SHR. In SHR with LV hypertrophy (15 weeks of age), the peak early filling rate of LV was unchanged, but the peak late filling rate was reduced by 25%, suggesting impaired atrial filling of LV [148]. Contractile function (sarcomere shortening) in atrial myocytes was unchanged during compensated LV hypertrophy but greatly decreased when SHR exhibited signs of heart failure [103]. Larger animal models of hypertension also exhibited reduced LA contractile function and LA myocyte shortening [43,121]. In the general population, decreasing LA emptying fraction (LAEF) was associated with hypertension and, independently, with mortality [106]. On the other hand, Ran and colleagues reported that hearts of patients with hypertension but no LV hypertrophy showed increased LA contractile function versus normotensive control subjects [149]. A potential explanation for this observation is that neurohumoral activation and atrial stretch, which are very early events in essential hypertension, induce positive inotropic effects in the atrial myocardium. A positive inotropic effect has been shown, for example, for noradrenaline, AngII, ET-1 and stretch in isolated human atrial myocardium [75,85,88,150,151]. In advanced hypertension with LV hypertrophy, LA remodeling such as fibrosis and altered responsiveness to inotropic stimuli [152] might counteract or attenuate these inotropic effects.

Nevertheless, in animal models and in patients with chronic hypertension and structural remodeling, impaired LA contractile function is a consistent finding. What might be the consequences of reduced LA contractility? Since LA contraction contributes to LV filling and cardiac output, reduced LA function will impair LV function and reduce cardiac output. While this might go unrecognized and not matter much in a healthy heart, it might be critical for cardiac function in a diseased heart with already compromised LV function. In line with this, in hypertensive patients with increased risk for LV diastolic dysfunction, reduced LA contractile function was a predictor for adverse cardiac outcomes and death [71]. Similarly, in patients with heart failure, increasing LAEF was independently associated with survival [153]. There are strong associations between hypertension, HFpEF and LA dysfunction [154]. In patients with HFpEF, LA dysfunction is associated with the onset of symptoms and may occur even in the absence of LA enlargement [154,155,156]. LA dysfunction may be a marker of the severity of HFpEF [155].

### 6.2. Atrial Fibrillation

Hypertension is a well-known risk factor for AF [2,7,9,157]. The hypertension-mediated LA remodeling not only impairs LA contractile function, it also increases the propensity for the development and perpetuation of AF. Structural remodeling (LA enlargement, fibrosis) along with altered conduction velocity/heterogeneity and LAERP create the substrate for AF contributing to the maintenance of the arrhythmia, while altered Ca^2+^ handling may serve as the trigger initiating the first episodes of AF. In addition, neurohumoral activation (via IP_3_ signaling) and acute atrial stretch might also trigger AF via increasing the propensity of spontaneous Ca^2+^ release events in atrial myocytes [55,158]. 

Thus, in animal models of hypertension, there is an increased likelihood for inducible atrial tachyarrhythmias. This has been shown for SHR [104,105,110] as well as for larger animal models [43,120,121,122]. In SHR, the likelihood and the duration of induced AF episodes increased with increasing severity of LA remodeling and age [104,105]. Moreover, even spontaneously occurring episodes of atrial tachyarrhythmias were observed in SHR in an age-dependent manner [24]. 

What are the consequences of AF in hypertension? Because in AF the atria stop contracting, the loss of LA contractions will cause the same chain of events as described above for reduced LA contractility. In AF with a high ventricular rate, there is tachycardia-induced cardiomyopathy [159]. A most recent study also showed that AF, even when the rate is controlled, causes LV remodeling with altered excitation–contraction coupling and ion homeostasis, including prolonged APD, reduced Ca^2+^ transients and SR Ca^2+^ load and elevated [Na^+^]_i_ [160]. Thus, there are multiple mechanisms by which AF can impair LV function. Accordingly, there are strong associations between LA dysfunction, AF and heart failure, in particular HFpEF [153,154,161,162,163].

**Table 1:** Remodeling in some frequently used animal models of hypertension with a particular focus on atrial remodeling.

		Animal Models of Hypertension				
Remodeling	Feature	SHR	AngII	L-NAME	DSS	DOCA
**Macroscopic** **alterations**	Maximum SBP	~200 mmHg [15]	~200 mmHg [164]	~200 mmHg [40]	~220 mmHg [26]	p: ~140 mmHg [42] r: ~180 mmHg [41]
	LA pressure	>4–5 Mo ↑ [73]	?	?	?	?
	LA dilatation P-wave duration	HF stage [105,165] >15 Mo ↑ [23]	m: 3 wkT ↑ [84,166]	6 wkT ↑ [167]	13 wkT [28]	r: 5 wkT [41] p: 3 MoT [42]
	AERP	11 Mo (~) [104] >12 Mo ↓ [105]	m: 3 wkT ↑ [84]	?	?	p: 3 MoT (~) [43]
	Atrial conduction velocity: slow/heterogeneous	>9 Mo [105,110]	m: 3 wkT [84]	?	?	?
	AF inducibility	>6 Mo [104,105,110,119]	m: 3 wkT [142]	?	>6 wkT [28]	r: old [168] p: 3 MoT [43]
	LA contractility	>3.5 Mo [148], >18 Mo ↓ [165]	?	?	?	p: 3 MoT ↓ [43]
**Structural**	Interstitial fibrosis	>3 Mo ↑ [104,109,111,119]	r: 2 wkT ↑ [169] m: 3 wkT ↑ [84]	>2 wkT ↑ [40,170]	>6 wkT [28]	p: 3 MoT↓ [42]
	Myocyte hypertrophy	>12 Mo [110]	m: 3 wkT [171]	?	>6 wkT [28]	p: 3 MoT [42]
	Apoptosis/Necrosis	>12 Mo [172]	r: 2 wkT [169]	?	?	?
**Electrical**	Action potential duration	6 Mo ↑ [119] 3–12 Mo (~) [104,110]	m: 3 wkT ↑ [84]	?	?	?
	I_Na_	>6 Mo I_NaL_ ↑ [125]	m: 3 wkT ↓ [84]	?	?	?
	I_K_	6 Mo ↓ [119], 11 Mo ↑ [104]	m: 3 wkT ↓ [84]	?	?	?
	I_CaL_/Cav1.2	>3 Mo up to HF ↓/↓ [103,104,109]	?/m: 3 wkT (~) [173]	?	?	?
	I_NCX_/NCX	6 Mo ↓/(~) [109]	?	?	?	p: 3 MoT (~) [43]
**Calcium**	Ca^2+^ transients	>3 Mo up to HF (~) [103,109]	m: 3 wkT (~) [142]	?	?	p: 3 MoT ↓ [43]
	SR Ca^2+^ load/FCR	6 Mo, HF ↑/↓ [103,109]	m: 3 wkT (~) [142]	?	?	p: 3 MoT ↓/(~) [43]
	SERCA	>3 Mo (~); >14 Mo/HF ↓ [103,109]	?	?	?	p: 3 MoT (~) [43]
	RyR2 expression/ RyR2 phosphorylation	>6 Mo ↓/↑ PKA [109] >14 Mo/HF ↓/↓ CaMKII [103]	?	?	?	?
	Myocyte contractility	HF ↓ [103]	?	?	?	p: 3 MoT ↓ [43]
**Autonomic** **regulation**	SNS hyperactivity	>1 Mo [64]	m: 3 wkT [164]	8 wkT (~) [174]	4 Mo [175]	r: 7 wkT ↑ [176]
	SNS/PNS imbalance	12 Mo [24]	m: 3 wkT [166]	up to 8 wkT (~) [170]	1.5 Mo [177]	?
**Humoral** **mediators**	Plasma ANP levels	(~) [96]	?	4 wkT ↑ [178]	>1 Mo ↑ [179]	r ↑ [180]
	Systemic RAAS	4 Mo ↑ [181]	-	6 wkT ↑ [182]	13 wkT ↓ [28]	r: 7 wkT ↑ [176]
	Atrial NA levels	1 Mo ↑ [64]	?	6 wkT (~) [182]	4 Mo ↓ [175]	r: 4 wT ↓ [183]
	Atrial AngII levels	8 Mo ↑ [81]	-	?	?	?
	Atrial ET-1 levels	8 Mo ↑ [81]	?	?	?	r: ↑ [184]
**Atrial** **inflammatory** **profile**	Macrophage infiltration	12 Mo > 15 Mo [105]	m: 3 wkT [173]	?	13 wkT [28]	?
	Pro-inflammatory cytokines	>5 Mo ↑ [81,119,144]	m: 3 wkT ↑ [173]	↑ heart [185]	13 wkT ↑ [28]	?
	Pro-fibrotic cytokines (TGFβ)	>4 Mo ↑ [81,111,119,144]	m: 3 dT ↑, m: 3 wkT (~) [171] or ↑ [173]	8 wkT ↑ [40,185]	?	?
	NLRP3 inflammasome	5 Mo ↑ [144]	?	?	?	?
	Oxidative stress	>5 Mo [81,119]	m: 3 wkT [142]	↑ heart [185]	13 wkT [28]	p: 3 MoT [42]

Unless mentioned otherwise, all levels of remodeling refer to atrial tissue. Animal models arranged (from left to right) according to the number of publications (high to low). AERP = atrial effective refractory period; AF = atrial fibrillation; AngII = hypertension induced by chronic infusion of angiotensin II; DOCA = 11-deoxycorticosterone acetate; DSS = Dahl salt-sensitive rat; dT = days of treatment; ET-1 = endothelin 1; I_Ca,L_ = L-type calcium current; I_K_ = potassium current; I_Na_ = sodium current; I_NCX_ = Na/Ca exchanger current; LA = left atrium; L-NAME = hypertension induced by long-term oral administration of N^ω^-nitro-L-arginine methyl esther (L-NAME) (rat); MAP = mean arterial pressure; Mo = months of age; MoT = months of treatment; NA = noradrenaline; NLRP3 = nucleotide-binding oligomerization domain (NOD)-like receptor containing pyrin domain 3; PNS = parasympathetic nervous system; RAAS = renin angiotensin aldosterone system; RyR2 = ryanodine receptor type 2; SR = sarcoplasmic reticulum; SERCA = sarcoplasmic/endoplasmic reticulum calcium ATP-ase; SHR = spontaneously hypertensive rat model; SNS = sympathetic nervous system; TGFβ = transforming growth factor β; wkT = weeks of treatment; (~) unchanged; ↓ decreased; ↑ increased level or activity; ? = no study found; m = mouse; p = pig; r = rat.

In summary, LA remodeling in hypertension results in LA contractile dysfunction and AF, which can cause LV dysfunction and heart failure (Figure 1).

## Abbreviations

AC: adenylyl cyclase(s); ACE, angiotensin-converting enzyme; ACh, acetylcholine; AERP, atrial effective refractory period; AF, atrial fibrillation; cAMP, cyclic adenosine monophosphate; AngII, angiotensin II; ANP, atrial natriuretic peptide; ANS, autonomic nervous system; AP, action potential; APD, action potential duration; ARB, Angiotensin II type 1 receptor blockers; BNP, B-type natriuretic peptide; CaMKII, Ca^2+^/calmodulin-dependent protein kinase II; CaTs, [Ca^2+^] transients; CV, conduction velocity; Cx, connexin; CTGF, connective tissue growth factor; DADs, delayed afterdepolarizations; DOCA, 11-deoxycorticosterone acetate; DSS, Dahl salt-sensitive rat(s); EADs, early afterdepolarizations; ECG, electrocardiogram; ET-1, endothelin-1; FHH, fawn-hooded hypertensive rat(s); HF, heart failure; HFpEF, heart failure with preserved ejection fraction; HFrEF, heart failure with reduced ejection fraction; I_Ca,L_, L-type calcium current; IL, interleukin; IP_3_, inositol 1,4,5-trisphosphate; L-NAME, N^ω^-nitro-L-arginine methyl ester; LA, left atrial; LAA, left atrial appendage; LV, left ventricular; LVEDP, left ventricular end-diastolic pressure; MAP, monophasic action potential; NA, noradrenaline; NCX, Na^+^/Ca^2+^ exchanger; NFAT, nuclear factor of activated T cells; NFκB, nuclear factor kappaB; NLRP3, nucleotide-binding oligomerization domain (NOD)-like receptor containing pyrin domain 3; PKA, protein kinase A; PLB, phospholamban; PNS, parasympathetic nervous system; RAAS, renin-angiotensin-aldosterone system; ROS, reactive oxygen species; RyR, ryanodine receptor(s); SAC, stretch-activated channel; SACs, stretch-activated channels; SERCA, sarcoplasmic/endoplasmic reticulum Ca^2+^ ATPase; SHR, spontaneously hypertensive rat(s); SNS, sympathetic nervous system; SR, sarcoplasmic reticulum; TGFβ, transforming growth factor β; TLR, toll-like receptors; TNFα, tumor necrosis factor α; WKY, Wistar-Kyoto rat(s).
